# Plant viruses of the *Amalgaviridae* family evolved via recombination between viruses with double-stranded and negative-strand RNA genomes

**DOI:** 10.1186/s13062-015-0047-8

**Published:** 2015-03-29

**Authors:** Mart Krupovic, Valerian V Dolja, Eugene V Koonin

**Affiliations:** Department of Microbiology, Unité Biologie Moléculaire du Gène chez les Extrêmophiles, Institut Pasteur, Paris, 75015 France; Department of Botany and Plant Pathology, Oregon State University, Corvallis, OR 97331 USA; National Center for Biotechnology Information, National Library of Medicine, National Institutes of Health, Bethesda, MD 20894 USA

**Keywords:** *Amalgaviridae*, Virus origin, dsRNA viruses, Negative-sense RNA viruses, Capsid proteins

## Abstract

Plant viruses of the recently recognized family *Amalgaviridae* have monopartite double-stranded (ds) RNA genomes and encode two proteins: an RNA-dependent RNA polymerase (RdRp) and a putative capsid protein (CP). Whereas the RdRp of amalgaviruses has been found to be most closely related to the RdRps of dsRNA viruses of the family *Partitiviridae*, the provenance of their CP remained obscure. Here we show that the CP of amalgaviruses is homologous to the nucleocapsid proteins of negative-strand RNA viruses of the genera *Phlebovirus* (*Bunyaviridae*) and *Tenuivirus.* The chimeric genomes of amalgaviruses are a testament to the effectively limitless gene exchange between viruses that shaped the evolution of the virosphere.

**Reviewers:** This article was reviewed by Lakshminarayan M. Iyer and Nick V. Grishin. For complete reviews, see the Reviewers’ Reports section.

**Electronic supplementary material:**

The online version of this article (doi:10.1186/s13062-015-0047-8) contains supplementary material, which is available to authorized users.

## Findings

Eukaryotes host a variety of RNA viruses which have positive-strand genomes (virions contain RNA of the same polarity as mRNA), negative-strand genomes (virions contain RNA complementary to the mRNA) or dsRNA genomes [1,2]. These viruses prey on hosts from all major eukaryotic supergroups and are extremely diverse both genetically and structurally, making it difficult to trace their origins. Genetic recombination, which often leads to the emergence of new chimeric entities, dramatically complicates the reconstruction of the evolutionary history of RNA viruses [3,4]. One approach to delineate the underlying evolutionary relationships is based on phylogenetic analysis of viral RNA-dependent RNA polymerases (RdRp), the only protein universally present in all non-defective RNA viruses [2,5]. A complementary strategy focuses on comparison of viral proteins responsible for virion formation, the hallmark of viruses [6-11]. It is becoming increasingly clear that only a combination of the two approaches can capture the full extent of evolutionary connections among different groups of viruses and other types of selfish genetic elements [12,13].

The evolution of viruses with dsRNA genomes appears to be particularly convoluted. In all likelihood, this class of viruses is polyphyletic: some groups appear to have evolved from different lineages of eukaryotic positive-strand RNA viruses, whereas others have apparently emerged from dsRNA bacteriophages [5,14-18]. Recently, a new group of dsRNA viruses has been described and classified into the family *Amalgaviridae*. These viruses have been isolated from various plants in the form of dsRNA molecules of 3.5 kb [19-22]. The genomes uniformly encode two predicted proteins, the RdRp and a putative capsid protein (CP). Such bicistronic genome organization is characteristic of dsRNA viruses of the family *Totiviridae* which infect fungi and protists [23]. However, phylogenetic analyses have shown that the amalgavirus RdRps form a sister clade to the corresponding proteins of partitiviruses (*Partitiviridae*) which have segmented (bipartite) dsRNA genomes and infect plants, fungi and protists (Additional file 1: Figure S1) [20-22]. Consequently, it has been suggested that amalgaviruses “amalgamate” features of totiviruses and partitiviruses (hence the family name) and represent an intermediate between the two viral groups [20]. However, both totiviruses and partitiviruses form icosahedral virions [23,24], whereas in the case of amalgaviruses, all attempts to visualize virus particles have so far failed [20-22]. This result was suggested to signify either low titer of amalgaviral particles or lack of bona fide viral particles altogether, as is the case for hypoviruses and endornaviruses [22]. Indeed, the putative CP of amalgaviruses shows no significant sequence similarity to the CPs of totiviruses or partitiviruses, and sequence analyses of this protein previously offered no clues as to its provenance. Immuno-gold labeling has shown that the putative CP is expressed during viral infection and is found within amorphous bodies in the cytoplasm [25]. Here we trace the source of the putative amalgaviral CP to negative-strand RNA viruses and propose an evolutionary scenario for the origin of these unusual dsRNA viruses.

BLASTp searches seeded with sequences of CPs from the four currently known amalgaviruses, Southern tomato virus (STV [22]), Vicia cryptic virus M (VCV-M [19]), Blueberry latent virus (BBLV [20]), and Rhododendron virus A (RdVA [21]), did not reveal any relationship to proteins of other viruses, consistent with previous attempts. However, several CP homologs were detected in the genomes of various plants, including *Populus trichocarpa*, *Medicago truncatula* and *Theobroma cacao*. In *P. trichocarpa*, the CP homolog was found next to a truncated BBLV-like RdRp gene (XP_002310263 matches both CP [E=6e-09] and RdRp [E=7e-25] of BBLV), pointing towards occasional, probably spurious integration of amalgavirus sequences into the host DNA and suggesting that the host range of amalgaviruses might be considerably broader than currently known.

**Figure 1:**
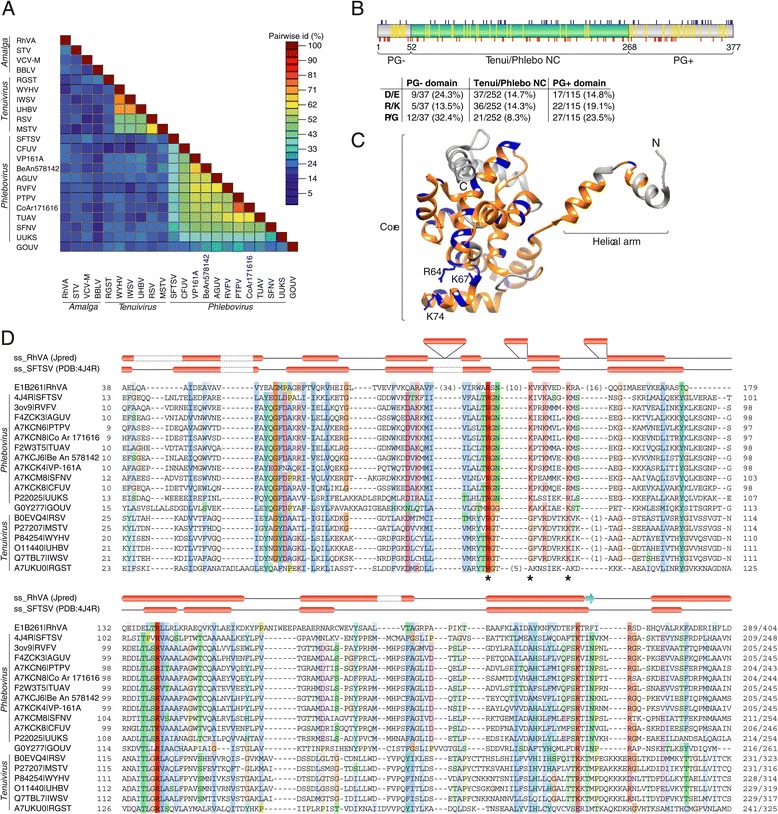
**Relationship between the putative capsid proteins of amalgaviruses and nucleocapsid proteins of tenuiviruses and phleboviruses. A**. Pairwise identity plot of capsid and nucleocapsid proteins of amalgaviruses, tenuiviruses and phleboviruses. Protein accession numbers are provided in panel D, whereas the full virus names are provided in the Abbreviations section. **B**. Schematic representation of the putative capsid protein of rhododendron virus A (RhVA). The proline/glycine-rich N- and C-terminal domains (PG- and PG+, respectively) are shown in grey, whereas the central domain showing similarity to the nucleocapsid proteins of phleboviruses and tenuiviruses (Tenui/Phlebo NC) is in green. Blue and red ticks above and below the scheme indicate the positions of positively (Arg, Lys) and negatively (Asp, Glu) charged amino acid residues, respectively, whereas yellow streaks correspond to Pro and Gly residues. The table includes the counts and percentages of Asp/Glu (D/E), Arg/Lys (R/K) and Pro/Gly (P/G) residues in the respective domains. **C**. X-ray structure of the nucleocapsid protein of Severe fever with thrombocytopenia syndrome virus (SFTSV). Amino acid residues shared with RhVA CP are shown in blue, regions that have counterparts in RhVA but do not show close sequence similarity are shown in orange, and regions that are absent in the RhVA protein are shown in grey. The residues important for RNA-binding are shown with ball-and-stick representation. The ‘Core’ and ‘Helical arm’ domains are indicated. **D**. Multiple sequence alignment of RhVA CP with tenuiviral and phleboviral NC homologs. Sequences are identified with UniProt or PDB accession numbers followed by abbreviated virus names. Above the alignment are the predicted (for RhVA) and experimentally determined (for SFTSV) secondary structure elements; α-helices, red ellipses; β-strands, blue arrows. The alignment is colored according to sequence conservation using the standard Clustal color scheme.

Amalgaviral CPs are highly divergent and share only 19-25% sequence identity (with the closest pair being RhVA–STV; Figure 1A). The common feature of these proteins is high content of predicted α-helices and near complete absence of β-strands [20-22]. To search for remote homologs of amalgaviral CPs, we employed HHpred [26] which performs pairwise comparison of hidden Markov model profiles and takes into account the experimentally determined or predicted secondary structures of the compared proteins. Unexpectedly, searches seeded with the putative CP of STV returned hits to the nucleocapsid (NC) proteins of animal-infecting phleboviruses (genus *Phlebovirus*, family *Bunyaviridae*) and plant tenuiviruses (unassigned genus *Tenuivirus*). Both phleboviruses and tenuiviruses have segmented negative-sense RNA genomes that are complexed with the NC proteins to form ribonucleoprotein (RNP) filaments [27,28]. Whereas phleboviral RNPs are packed into spherical membrane-bound virions, the RNPs of tenuiviruses are not further encapsidated and persist as non-enveloped filamentous structures that can adopt circular or branched configurations [28]. Structural analyses of NC proteins from several phleboviruses have revealed a unique α-helical fold consisting of two domains: the core domain involved in sequence unspecific RNA binding and the flexible helical arm domain responsible for protein oligomerization [29-31]. The NC is unique to viruses from these two genera and thus far has not been reported for any other virus group. Besides the NC, phleboviruses and tenuiviruses share several other proteins, and in phylogenetic analyses of the RdRps, tenuiviruses cluster with phleboviruses, deeply within the family *Bunyaviridae*, suggesting that tenuiviruses have evolved from phleboviruses [28].

The region matched by HHpred between STV CP and phleboviral/tenuiviral NCs, extended over 216 residues (from 52 to 266) of the STV protein (Figure 1B) and encompassed most of the phleboviral NC protein sequence (197 of 248 residues). Although statistical significance of the obtained hits was not particularly high (P=71.8%; Additional file 2: Figure S2), we pursued this lead and investigated the relationship in more detail. To validate the potential homology of amalgaviral CPs and phleboviral/tenuiviral NCs, a representative set of phleboviral/tenuiviral NC sequences was downloaded from the PFAM database (Family: *Tenui_N*; PF05733) and aligned with the proteins of the four amalgaviruses. Notably, tenuiviral NC sequences were as similar to the phleboviral homologs as they were to amalgaviral CPs (Figure 1A). Due to the high divergence of amalgaviral CPs, multiple sequence alignments were constructed between phleboviral/tenuiviral NCs and individual CPs of amalgaviruses. As can be judged from the sequence alignment shown in Figure 1D, amino acid positions conserved between tenuiviruses and phleboviruses are also conserved in the CP of amalgavirus RhVA. Furthermore, the predicted secondary structure profile of the RhVA CP is strongly similar to the experimentally determined structure of the NC of Severe fever with thrombocytopenia syndrome virus (*Phlebovirus*), despite several insertions in the former protein (Figure 1D). It should be noted that, the high sequence divergence notwithstanding, the secondary structure profiles are closely similar for all four amalgaviral CPs (Additional file 3: Figure S3). Analysis of the sequence conservation distribution in the context of the tertiary structure of the phleboviral NC shows that the conserved residues are evenly distributed within the ‘core’ domain of the protein (Figure 1C). Notably, the three positively charged residues, which have been found to play an essential role in RNA binding by the Rift Valley fever virus NC [29], are conserved in the RhVA protein. However, certain degree of variation is observed in this region in CPs of other amalgaviruses (Additional file 3: Figure S3), as is the case for tenuiviral NCs (Figure 1D).

The major differences between the amalgaviral CPs both within the family (Additional file 3: Figure S3) as well as when compared to the phleboviral/tenuiviral NCs are localized to the N and C termini. In particular, RhVA CP, which is the largest among amalgaviral CPs (404 aa), lacks the regions corresponding to the first of the two α-helices within the N-terminal helical arm domain and the two C-terminal α-helices (Figure 1C). The three α-helices (α1, α12 and α13 in Rift Valley fever virus NC) are located at the periphery of the hexameric NC ring, and residues projecting from these helices have been proposed to form a large part of the RdRp-binding surface [29]. Given that the RdRps of amalgaviruses and phleboviruses are highly dissimilar, it seems likely that the changes within the N- and C-terminal regions of the amalgaviral CPs were driven by the necessity to adapt to the partitivirus-like RdRp (Additional file 1: Figure S1). Both the N-terminal and the C-terminal regions in the RhVA CP are rich in proline and glycine residues. Furthermore, when compared to the central domain, which bears similarity to the phleboviral/tenuiviral NCs, the N-terminal region is enriched in negatively charged amino acids, whereas the C-terminal region is more positively charged (Figure 1B). Notably, the C-terminal region of the tenuiviral NC is also enriched in positively charged residues and has been shown to bind nucleic acids [32]. Consequently, although N- and C-terminal regions display considerable variation among the amalgaviral CPs (Additional file 3: Figure S3), they might play important roles in mediating various protein-protein and protein-RNA interactions.

## Conclusions

It has been proposed that amalgaviruses share features with partitiviruses and totiviruses and thus might represent an evolutionary link between the two virus groups [22]. Our results indicate that the CP of amalgaviruses evolved from the NC of phleboviruses or tenuiviruses. Considering that tenuiviruses replicate in plants, they appear as a more likely source of the NC gene of amalgaviruses. We propose that the ancestor of amalgaviruses has emerged by recombination between a partitivirus, which contributed the RdRp gene, and a tenuivirus which donated the NC gene. In partitiviruses, the RdRp is the sole gene encoded in one of the two genomic segments [24]. Thus, incorporation of the NC gene into such genomic segment would conceivably lead to size increase of the resultant chimeric genome, precluding its incorporation into the original partitivirus-sized capsids. An outstanding question is the exact role of the CP in amalgaviruses. Phleboviral/tenuiviral NCs are known to bind ssRNA [31], whereas the genomes of amalgaviruses are thought to consist of dsRNA. On the one hand, it cannot be ruled out that following the acquisition from tenuiviruses, the CPs have evolved the ability to bind dsRNA instead of ssRNA. Indeed, phleboviral NCs have an inherent ability to form diverse oligomers and bind both ssRNA and ssDNA [29,31], whereas NC of tenuiviruses can also bind dsDNA [32]. On the other hand, amalgaviral CPs might interact with the single-stranded form of the genome. Given that dsRNA is the necessary intermediate that is always produced upon replication of both ssRNA and dsRNA viruses, the dsRNA genomes of amalgaviruses isolated directly from the plant tissues might represent a replicative intermediate rather than the “encapsidated” form of the genome. Another possibility is that the CP of amalgaviruses was recruited for a function distinct from the original role of the tenuivirus NC. For example, the NC of plant-infecting rhabdoviruses, besides encapsidating the viral genomic RNA, is a component of the viroplasms [28,33]. Amorphous bodies immuno-labeled with antibodies against BBLV CP in the cytoplasm of BBLV-infected cells could actually represent such viroplasm-like structures. Efficient vertical transmission of the amalgaviruses through the seed, together with their inability to mechanically transmit from plant-to-plant [20-22], are compatible with the apparent lack of the bona fide extracellular virions that are normally required for the latter transmission route. Regardless of the precise roles of the NC-derived protein in virus reproduction, amalgaviruses represent a remarkable case of transfer of viral hallmark genes between widely different RNA viruses and together with similarly striking examples of gene exchange between viruses with RNA and DNA genomes [34-36], emphasize the ultimate modularity of the virosphere.

## Methods

Sequences of the putative capsid proteins of amalgaviruses were obtained from GenBank and their homologs in viral and cellular genomes were searched for using BLASTp [37]. Distant homology detection was performed using HHpred [26]. Sequences of phleboviral and tenuiviral nucleocapsid proteins (family: *Tenui_N*) were downloaded from the PFAM database (PF05733) and clustered to 80% sequence identity using BLASTclust at http://toolkit.tuebingen.mpg.de/blastclust. Protein sequences were aligned using Promals3D [38] and visualized using Jalview [39]. Secondary structure of the rhododendron virus A capsid protein was predicted using Jpred [40]. All-against-all pairwise identities were calculated using Sequence Demarcation Tool [41]. X-ray structure of the Severe fever with thrombocytopenia syndrome virus was downloaded from the Protein Data Bank (4J4R) [30] and rendered using UCSF Chimera [42]. Maximum likelihood phylogenetic analysis of RdRps was carried out by using PhyML 3.1 [43], with the JTT model of amino acid substitution, including a gamma law with 4 substitution rate categories, and an estimated proportion of invariable sites.

## Reviewers’ reports

### Reviewer 1: Lakshminarayan M. Iyer, National Center for Biotechnology Information, NIH, Bethesda

This is an interesting twist on the origins of the Amalgaviridae and proposes their derivation from a recombination between a partitivirus and a tenuivirus. The study hinges on the unification of the Amalgaviral Capsid proteins with the Nucleocapsid proteins of negative strand RNA viruses such as Tenuviruses and Pheloboviruses using Profile-profile searches. I can reproduce their search results, although searches only retrieves the NC proteins from some starting points and alignment queries do not retrieve NC proteins in profile-profile searches. The statistics is also very weak. I would have been very hesitant if I were to make this conclusion. This is primarily because in my experience profile-profile searches might often retrieve unrelated alpha-helical proteins at low confidence values when alpha-helical proteins are used as query and one could be biased by the names of the retrieved proteins. However, to credit the authors, they have compared conservation of sequence and structure and have considered the structure of the core to reach their conclusions with very convincing arguments. The paper is well written otherwise and can be published without any modifications.

Authors’ response: *We appreciate this expert assessment.*

### Reviewer 2: Nick V. Grishin, University of Texas Southwestern Medical Center, Dallas

In this interesting, thoroughly-executed and well-written study, the authors successfully trace evolutionary origins of one of the two protein encoded by Amalgaviridae. Using best available sequence-based methods augmented with expert analysis of alignments, they find homologs of this protein in better-studied viruses and thus predict its 3D structure. This prediction looks very reasonable and has several interesting biological ramifications. First, since the homologs of this protein are capsid-forming (or nucleic-acid-packaging) proteins, this homology provides additional evidence that Amalgaviridae possess a capsid protein. Second, this homology suggests the origin of these viruses by recombination of genomes of two different groups of viruses. Third, it implies that unusual alpha-helical capsids may be more widespread than previously thought. More generally, finding homologs for viral proteins is very difficult due to high numbers of fixed amino acid substitutions leading to large sequence divergence between homologs. This is a well-known problem, and it is addressed very nicely in this paper. I.e., a careful study of weekly similar sequence or threading hits could be very productive.

Authors’ response: *We appreciate the expert appraisal of the article*.

Minor technical suggestions:

“recently established family” is not very clear, it may be understood as a family that originated recently in evolution. Maybe something like “recently described” or “recently erected” or smth similar.

Authors’ response: *good point*, ‘*established*’ *changed to* ‘*recognized*’.

Due to its more speculative nature, should this section be called “Discussion” instead?

Authors’ response: *We appreciate the suggestion but the Discovery Notes format mandates***Conclusions***following the***Findings***section. Hopefully, this is interpreted as intended, i.e. as “Discussion and Conclusions”.*

## Additional files

Additional file 1: Figure S1. Maximum likelihood tree of the RdRp proteins from diverse RNA viruses. The tree was rooted on the branch of positive-strand RNA viruses (Picornaviridae and Caliciviridae). Numbers at the branch points represent SH-like local support values. Branches with support values below 50% were collapsed. The scale bar represents the number of substitutions per site. Additional file 2: Figure S2. Results of the HHpred search seeded with the putative capsid protein of Southern tomato virus (YP_002321510). H(h), α-helix; C(c), coil. Additional file 3: Figure S3. Multiple sequence alignment of amalgaviral capsid proteins. All sequences are indicated with their GenBank identifiers followed by abbreviated virus names. Positively charged amino acid residues predicted to be involved in RNA binding are shown in bold and underlined. The last two lines in each block show consensus amino acid sequence (Consensus_aa) and consensus predicted secondary structures (Consensus_ss). The protein sequences are colored according to predicted secondary structures (red: alpha-helix, blue: beta-strand). Consensus predicted secondary structure symbols: alpha-helix: h; beta-strand: e. Consensus amino acid symbols are: conserved amino acids are in uppercase letters; aliphatic (I, V, L): l; aromatic (Y, H, W, F): @; hydrophobic (W, F, Y, M, L, I, V, A, C, T, H): h; alcohol (S, T): o; polar residues (D, E, H, K, N, Q, R, S, T): p; tiny (A, G, C, S): t; small (A, G, C, S, V, N, D, T, P): s; bulky residues (E, F, I, K, L, M, Q, R, W, Y): b; positively charged (K, R, H): +; negatively charged (D, E): −; charged (D, E, K, R, H): c. The alignment was constructed with PROMALS3D (http://prodata.swmed.edu/promals3d).

## Abbreviations

AGUV: Aguacate virus
Be An 578142: Phlebovirus sp. Be An 578142
CP: Capsid protein
CFUV: Corfou virus
Co Ar 171616: Phlebovirus sp. Co Ar 171616
dsRNA: Double-stranded RNA
GOUV: Gouleako virus (unclassified bunyavirus)
IWSV: Iranian wheat stripe virus
MSTV: Maize stripe virus
NC: Nucleocapsid
PTPV: Punta toro phlebovirus
RGST: Rice grassy stunt virus
RSV: Rice stripe virus
RdRp: RNA-dependent RNA polymerase
SFNV: Sandfly fever Naples virus
TUAV: Turuna virus
UHBV: Urochloa hoja blanca virus
UUKS: Uukuniemi virus
VP-161A: Phlebovirus sp. VP-161A; WYHV: Wheat yellow head virus
