# Supplementary material for: Plant viruses of the Amalgaviridae family evolved via recombination between viruses with double-stranded and negative-strand RNA genomes
Source: Biol Direct. 2015 Mar 29;10:12. doi: 10.1186/s13062-015-0047-8 (PMC4377212; doi:10.1186/s13062-015-0047-8)
Supplement: Additional file 1: Figure S1. — Maximum likelihood tree of the RdRp proteins from diverse RNA viruses. The tree was rooted on the branch of positive-strand RNA viruses (Picornaviridae and Caliciviridae). Numbers at the branch points represent SH-like local support values. Branches with support values below 50% were collapsed. The scale bar represents the number of substitutions per site. [file 13062_2015_47_MOESM1_ESM.pdf]

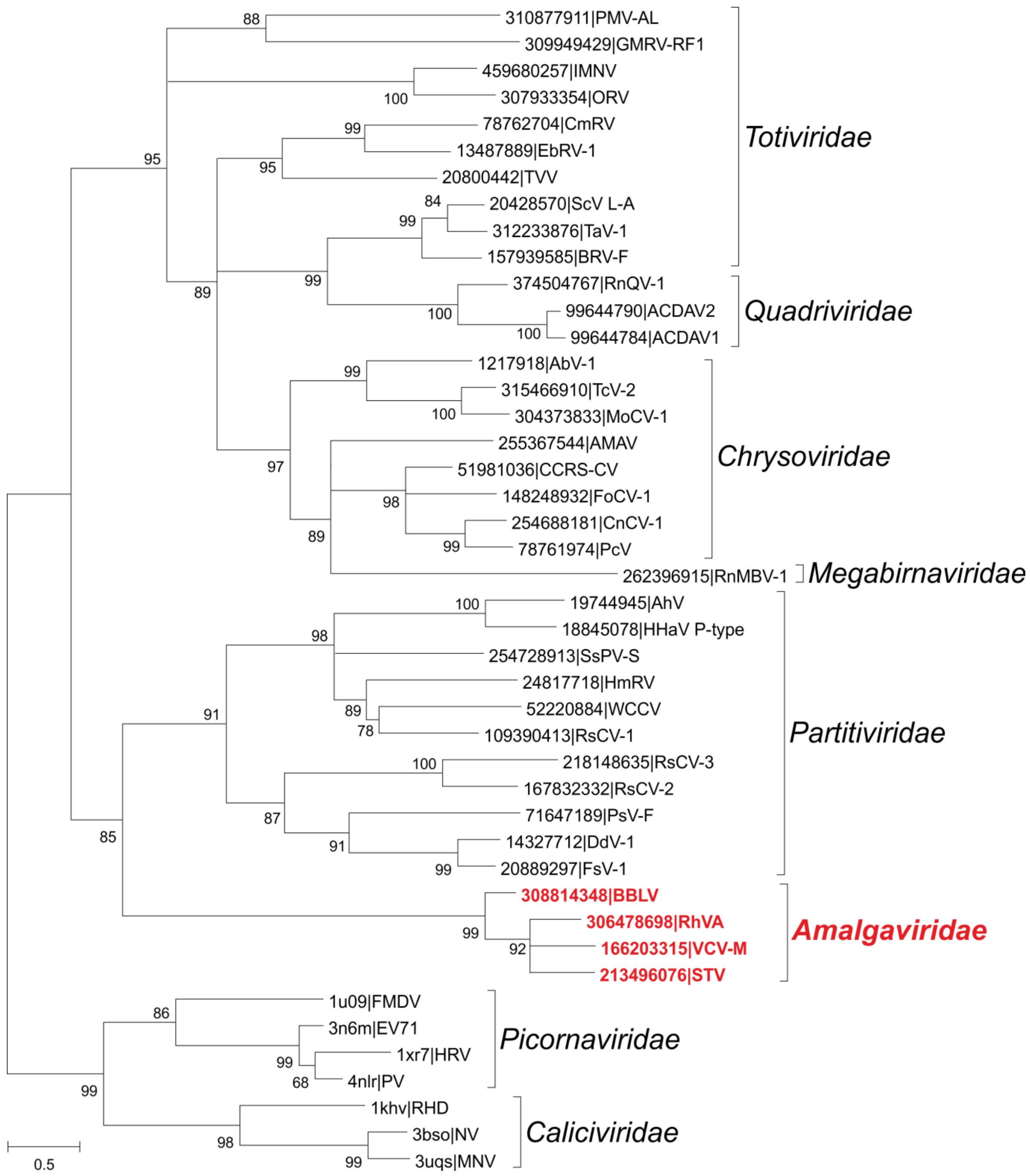

Figure S1. Maximum likelihood tree of the RdRp proteins from diverse RNA viruses. The tree was rooted on the branch of positive-strand RNA viruses (*Picornaviridae* and *Caliciviridae*). Numbers at the branch points represent SH-like local support values. Branches with support values below 50% were collapsed. The scale bar represents the number of substitutions per site.
