# Supplementary material for: Plant viruses of the Amalgaviridae family evolved via recombination between viruses with double-stranded and negative-strand RNA genomes
Source: Biol Direct. 2015 Mar 29;10:12. doi: 10.1186/s13062-015-0047-8 (PMC4377212; doi:10.1186/s13062-015-0047-8)
Supplement: Additional file 2: Figure S2. — Results of the HHpred search seeded with the putative capsid protein of Southern tomato virus (YP_002321510). H(h), α-helix; C(c), coil. [file 13062_2015_47_MOESM2_ESM.pdf]

>4j4r_A SFTSVN, nucleocapsid protein; nucleoprotein, viral Pro; 1.90A {Severe fever with thrombocytopenia syndrome virus; *Phlebovirus*} PDB: 4j4u_A 4j4v_A* 4j4s_A
Probab=71.76 E-value=2.2e+02 Score=30.57 Aligned_cols=167 Identities=16% Similarity=0.058 Sum_probs=0.0

```
Q ss_pred              HHHHHHccCCHHHHHHHHHHHHcC--ccHHHHHHHHHHHHHHHHHHHhhhcCCHHHHHHHHHHhcChHHHHHHHHHHHHHHH
Q Fri_Jan_23_09:   52 RTTYVSHMLLPNQYFKLLKQFKG--KTAEELDLALGAAVAHGVLRSMRGITFKKFFDFLNWVKTKEGKDALGETMYAQKL  129 (377)
Q Consensus        52 v~~~~~~~Ltv~~F~k~iK~~~g------eld~i~~~~v~~Gv~~s~~~~tlkKff~f~rWl~T~~G~~aL~~~qkqkkL  129 (377)
                      ++.+--+|+.+...++.+|.-+|  -..|-...|        |+.-.|+..|++   ...-. +++|+..+....++=+|
T Consensus        28 v~~f~YqGFDa~~ii~~lkerGg~~~~~D~k~mI--------Vl~LtRGnk~~K---m~~km-s~~Gk~~v~~Li~~Y~L   95 (248)
T 4j4r_A           28 ARELAYEGLDPALIIKKLKETGGDDWVKDTKFII--------VFALTRGNKIVK---ASGKM-SNSGSKRLMALQEKYGL   95 (248)
T ss_dssp              HHHHSCCCCCHHHHHHHHHHHHGGGHHHHHHHHH--------HHHHHHTTCHHH---HHHTS-CHHHHHHHHHHHHHHHTB
T ss_pred              HHHHHhcCCCHHHHHHHHHHHhhhHHHHHHHHHH--------HHHHhhCCcHHH---HHHHh-hHhhHHHHHHHHHHHhCe


Q ss_pred              Hhc--ccccCCHHHHHHHHHHHHHHHHHHHHHHHHHHhhHHHHHHHHHHHHHHHHHHHHHHHHHHHHHHHHHHhcCch---------
Q Fri_Jan_23_09:  130 EQK--GRGDFSIAEVALLHCFETQRNDMLRDEKDVRLKAEEEIADLQRKIVKRREKLEEDLIATKSNYEPV---------  198 (377)
Q Consensus       130 e~k--g~g~~t~aqVaL~~lf~~qq~dyA~~~k~ar~~~~~EIaeL~rki~~~r~ele~~~~~lk~ky~P~---------  198 (377)
                      +.+  ++-++|-.-|+-|.-.-+-|.-...                                  ++.+|+.
T Consensus        96 ks~~p~r~~lTLsRVAa~fp~~t~q~~~~v-----------------------------------~~~lpV~~~~m~~~~~  141 (248)
T 4j4r_A           96 VERAETRLSITPVRVAQSLPTWTCAAAAAL-----------------------------------KEYLPVGPAVMNLKVE  141 (248)
T ss_dssp              CSSCCSTTCBCHHHHHHHSHHHHHHHHHHT-----------------------------------GGGCSSSHHHHHHHSS
T ss_pred              ecCCCCcCcccHHHHHHHchHHHHHHHHHH-----------------------------------HhhcCcccccccccc


Q ss_pred              -----------hcccc-----ccHHHHHHHHHHHHHhhhhhHHHhccCcccch--hHHHHHHHHHHHHHHHHHHHHHHHhcCcc
Q Fri_Jan_23_09:  199 -----------SRYVG-----LSDYELNCKCWSLYQQFNPDKVTAGAKPTRK--QVKEAFDMYAEFVAKTNRLEFLKHGN  260 (377)
Q Consensus       199 ------------~~Y~~~~~~~~~~~enEL~~~Cw~lY~~~~~~dc~rkgi~P~~k----keAve~Yg~~V~e~~l~ef~~~~~  260 (377)
                                 .+.--     .+.-++-+.|+++|+-+    .++=|.|.-+  +.+|.++-|..-..-..--.||.++.
T Consensus       142 ~YP~~MMh~sFagLI~~~~l~~e~~~~i~dAh~Lf~~~~~~~~Fs~tINp~~rg~s~~ev~~sf~~pm~Aa~~SsF~~~e~  217 (248)
T 4j4r_A          142 NYPPEMMCMAFGSLIPTAGVSEATTKTLMEAYSLWQDA----FTKTINVKMRGASKTEVYNSFRDPLHAAVNSVFFPNDV  217 (248)
T ss_dssp              SCCGGGSSTTGGGGCCSSSSCHHHHHHHHHHHHHHHHH----HHHHHCGGGTTCCHHHHHHHHHHHHHHHHHCSSSCHHH
T ss_pred              CCCHHHhhhhhccCCCCCCCHHHHHHHHHHHHHHHHHH----HHHHhChhhcCCCHHHHHHHHHhHHHHHHHHhccCCCHHH


Q ss_pred              HHHHHHHH
Q Fri_Jan_23_09:  261 VKDELQAF  268 (377)
Q Consensus       261 ~Ke~L~~y  268 (377)
                      |+.-|.+|
T Consensus       218 R~~~L~~~  225 (248)
T 4j4r_A          218 RVKWLKAK  225 (248)
T ss_dssp              HHHHHHHH
T ss_pred              HHHHHHHh
```

>3ov9_A Nucleoprotein; orthogonal bundle, viral genomic encapsidation, viral protein; 1.60A {Rift valley fever Virus; *Phlebovirus*} PDB: 3ouo_A 3lyf_A 4h5m_A 4h5o_A 4h5p_A 4h5q_A
Probab=62.71 E-value=2.1e+02 Score=30.72 Aligned_cols=175 Identities=18% Similarity=0.234 Sum_probs=0.0

```
Q ss_pred              HHHHHHccCCHHHHHHHHHHHHcC--ccHHHHHHHHHHHHHHHHHHHhhhcCCHHHHHHHHHHhcChHHHHHHHHHHHHHHH
Q Fri_Jan_23_09:   52 RTTYVSHMLLPNQYFKLLKQFKG--KTAEELDLALGAAVAHGVLRSMRGITFKKFFDFLNWVKTKEGKDALGETMYAQKL  129 (377)
Q Consensus        52 v~~~~~~~Ltv~~F~k~iK~~~g------eld~i~~~~v~~Gv~~s~~~~tlkdff~f~rWl~T~~G~~aL~~~qkqkkL  129 (377)
                      ++.+--+|+.+..-++.+|.-+|  -..|-...|        |+.-.|+..|+|...=+    +++|+..+....++=+|
T Consensus        25 v~~faYqGFDa~~ii~~lk~~Gg~~~~~D~k~mI--------Vl~LtRGNk~~km~~km----s~~Gk~~v~~Li~~Y~L   92 (245)
T 3ov9_A           25 VREFAYQGFDARRVIELLKQYGGADWEKDAKKMI--------VLALTRGNKPRRMMMKM----SKEGKATVEALINKYKL   92 (245)
T ss_dssp              HHHHSBCSCCHHHHHHHHHHHHGGGHHHHHHHHH--------HHHHHTCSCHHHHGGGS----CHHHHHHHHHHHHHHHTC
T ss_pred              HHHHHhcCCCHHHHHHHHHHHHccchHHHHHHHH--------HHHHhhCCCHHHHHHHHh----hHhhHHHHHHHHHHHhCc


Q ss_pred              Hhc--ccccCCHHHHHH------HHHHHHHHHHHHHHHHHHHHhhHHHHHHHHHHHHHHHHHHHHHHHHHHHHHHHHhcCc----
Q Fri_Jan_23_09:  130 EQK--GRGDFSIAEVAL------LHCFETQRNDMLRDEKDVRLKAEEEIADLQRKIVKRREKLEEDLIATKSNYEP----  197 (377)
Q Consensus       130 e~k--g~g~~t~aqVaL-------~~lf~~qq~dyA~~~k~ar~~~~~EIaeL~rki~~~r~ele~~~~~lk~ky~P----  197 (377)
                      +.+  ++-++|-+-|+-      |++...=..-+.+....-                         ..+-.+||+
T Consensus        93 ks~~p~r~~lTLsRVAaafp~~tcqa~~~v~~~lpV~~~~m-------------------------~a~~~~YP~~MMh  146 (245)
T 3ov9_A           93 KEGNPSRDELTLSRVAAALAGWTCQALVVLSEWLPVTGTTM--------------------------DGLSPAYPRHMMH  146 (245)
T ss_dssp              CSSSCCTTCCCHHHHHHHTHHHHHHHHHHHGGGSSSCHHHH--------------------------HHHSTTCCGGGSS
T ss_pred              ccCCCCccccccHHHHHHHhhhHHHHHHHHHHHhhCCcCCcccc--------------------------ccccccCchhhhc


Q ss_pred              --hhcccccc----HHHHHHHHHHHHhhhhhHHHhccCcccch--hHHHHHHHHHHHHHHHHHHHHHHHhcCccHHHHHHHH
Q Fri_Jan_23_09:  198 --VSRYVGLS----DYELNCKCWSLYQQFNPDKVTAGAKPTRK--QVKEAFDMYAEFVAKTNRLEFLKHGNVKDELQAF  268 (377)
Q Consensus       198 ----~Y~~~~~~~~~~~enEL~~~Cw~lY~~~~~~dc~rkgi~P~~k----keAve~Yg~~V~e~~l~ef~~~~~~Ke~L~~y  268 (377)
                        +.+...++    ..++-+.|+.+||-+    .++=|-|.-+  +.+|.++-|..-..--.--.||.++.++.-|.+|
T Consensus       147 psFagLI~~~l~~e~~~~i~~Ah~Lf~~~~~~~FS~tINp~~r~~s~~ev~~sf~~pm~Aa~~s~f~~~~R~~~L~~~  221 (245)
T 3ov9_A          147 PSFAGMVDPSLPGDYLRAILDAHSLYLLQ----FSRVINPNLRGRTKEEVAATFTQPMNAAVNSNFISHEKRREFLKAF  221 (245)
T ss_dssp              GGGGGGCCTTSCHHHHHHHHHHHHHHHHHH----HHHHHCTTSCSCCHHHHHHHHHHHHHHHHHTCSSSCHHHHHHHHHHT
T ss_pred              chhhhcCCCCCCHHHHHHHHHHHHHHHHHH----HHHHhCHhhccCCHHHHHHHHHhHHHHHHHHcCCCCHHHHHHHHHHh
```

```
>PF05733 Tenui_N Tenuivirus/Phlebovirus nucleocapsid protein. This family consists of several Tenuivirus and
  Phlebovirus nucleocapsid proteins. These are ssRNA viruses.
  Probab=57.90  E-value=4e+02  Score=28.35  Aligned_cols=187  Identities=13%  Similarity=0.099  Sum_probs=0.0

  Q ss_pred              HHHHHHccCCHHHHHHHHHHHc-C-ccHHHHHHHHHHHHHHHHHHhhhcCCHHHHHHHHHHhcChHHHHHHHHHHHHHHHH
  Q Fri_Jan_23_09:   52 RTTYVSHMLLPNQYFKLLKQFK-G-KTAEELDLALGAAVAHGVLRSMRGITFKKFFDFLNWVKTKEGKDALGETMYAQKL  129 (377)
  Q Consensus        52 v~~~~~~~~Ltv~~F~k~iK~~~-g-~~~~~eld~i~~~~v~~Gv~~s~~~~tlkdff~f~rWl~T~~G~~aL~~~qkqkkL  129 (377)
                         ++.+-=+|+.+...++.++.-. | .=.+.+..+.-++..||       +.+++...=+    +++|++.+....++=+|
  T Consensus        23 v~~f~YqGFDa~~ii~~l~~~g~g~~~~kD~k~mIVl~ltRG-------Nk~~Km~~km----s~~Gk~~v~~Li~~Y~L   91 (224)
  T pfam05733        23 VNQFAYQGFDAARILQLLKEKGSGDDWGKDVKMMIVLRLTRG-------NKPKKIEKKK----SAEGAEEVAELITRYGL   91 (224)
  T ss_pred              HHHHHHcCCCHHHHHHHHHHHhccccHHHHHHHHHHhhhhHhcC-------CcHHHHHHHh----cHhhhHHHHHHHHHHhCe


  Q ss_pred              Hh--cccc--cCCHHHHHHHHHHHHHHHHHHHHHHHHHHHHhhHHHHHHHHHHHHHHHHHHHHHHHHHHHHHHHhcCchhcccccc
  Q Fri_Jan_23_09:  130 EQ--KGRG--DFSIAEVALLHCFETQRNDMLRDEKDVRLKAEEEIADLQRKIVKRREKLEEDLIATKSNYEPVSRYVGLS  205 (377)
  Q Consensus       130 e~--kg~g-~~~t~aqVaL~~lf~~qq~dyA~~~k~ar~~~~~EIaeL~rki~~~r~ele~~~~~lk~ky~P~~~Y~~~~  205 (377)
                         +.  +..|  ++|-.-||-+.-..+-|.-...+.+..              +.-..|..- .......-+-|+..+=-+.
  T Consensus        92 ~s~~g~p~r~~iTLsRvaa~f~~~t~~~~~~v~~~lp--------------V~~~m~~~~~~yP~~mmhp~FagLI~~  156 (224)
  T pfam05733        92 VESVGNNARDAITLSRVAAAFAGWTCQALKVVSAKLA--------------VDGSTMDTS-GPDYPRWMMHPQFAGLIDE  156 (224)
  T ss_pred              eeccCCCcccccHHHHHHhhhHHHHHHHHHHHhhCC--------------cCcccccC-CCCCcHHHhcccccCCC


  Q ss_pred              H-----HHHHHHHHHHHhhhhhHHHhccCccch--hHHHHHHHHHHHHHHHHHHHhcCccHHHHHHHH
  Q Fri_Jan_23_09:  206 D-----YELNCKCWSLYQQFNPDKVTAGAKPTRK--QVKEAFDMYAEFVAKTNRLEFLKHGNVKDELQAF  268 (377)
  Q Consensus       206 e-----nEL~~~Cw~lY~~~~~~dc~rkgi~P~~k---~keAve~Yg~~V~e~~l~ef~~~~~~~Ke~L~~y  268 (377)
                         +     ...-..|+.+|+.+    .++-|-|.-+  +-.|.++.|.....-..--.||.++.||.-|.+|
  T Consensus       157 lp~~~~~~l~~aH~Lf~~~~~---fSktInp~~r~~~~~e~~~~~~~~~~m~aa~~ssfl~~~~Rr~~l~~~  222 (224)
  T pfam05733       157 TLPKNSNNALFDAHLLYLLQ----FSKTINPSLRKKEKNEMVKYFTQPLVAACSSSFLTDEKRRKLLEAF  222 (224)
  T ss_pred              cCCHHHHHHHHHHHHHHHHH----HHhhhChhhccCcHHHHHHHHHHHHHHHHHccccCCHHHHHHHHHHc
```

Figure S2. Results of the HHpred search seeded with the putative capsid protein of Southern tomato virus (YP_002321510). H(h), α-helix; C(c), coil.
