# Supplementary material for: Plant viruses of the Amalgaviridae family evolved via recombination between viruses with double-stranded and negative-strand RNA genomes
Source: Biol Direct. 2015 Mar 29;10:12. doi: 10.1186/s13062-015-0047-8 (PMC4377212; doi:10.1186/s13062-015-0047-8)
Supplement: Additional file 3: Figure S3. — Multiple sequence alignment of amalgaviral capsid proteins. All sequences are indicated with their GenBank identifiers followed by abbreviated virus names. Positively charged amino acid residues predicted to be involved in RNA binding are shown in bold and underlined. The last two lines in each block show consensus amino acid sequence (Consensus_aa) and consensus predicted secondary structures (Consensus_ss). The protein sequences are colored according to predicted secondary structures (red: alpha-helix, blue: beta-strand). Consensus predicted secondary structure symbols: alpha-helix: h; beta-strand: e. Consensus amino acid symbols are: conserved amino acids are in uppercase letters; aliphatic (I, V, L): l; aromatic (Y, H, W, F): @; hydrophobic (W, F, Y, M, L, I, V, A, C, T, H): h; alcohol (S, T): o; polar residues (D, E, H, K, N, Q, R, S, T): p; tiny (A, G, C, S): t; small (A, G, C, S, V, N, D, T, P): s; bulky residues (E, F, I, K, L, M, Q, R, W, Y): b; positively charged (K, R, H): +; negatively charged (D, E): −; charged (D, E, K, R, H): c. The alignment was constructed with PROMALS3D (http://prodata.swmed.edu/promals3d). [file 13062_2015_47_MOESM3_ESM.pdf]

```
Conservation:         9              5            5        5 5  5                 9         55     5
306478699|RhVA     1  MEDRRAEDRLDDDLPAGGGAPPPLRGLPPLPRAEPTDAELQAAIDEAVAVVYEAGMPAGRFTVQRVHEIGLTVEVFVKQARAVFGGKNV  89
308814347|BBLV     1  M--------------AGASG-----SILPVETTEQTQARVSTALAPLTGVL----GNIERFSVEAIINSNLTVDIFLKAIKSLVAYAQQ  66
213496075|STV      1  MA------GVGGSAAGRVPNAA----NVPLT-AKEKERTVMREIVEIGETFVELGIDKRYFQRTTYVSHMLLPNQYFKLLKQFKGKTA-  77
166203315|VCV-M    1  MA------QFETSDPANVGEGA---GIGGIRSKAEEQATVNQAMNAIVRLG----FPEDIFDIDALLERGFTAKSWCDYCRAITSITER  76
Consensus_aa:         M..............t.ss.........sl.....pp.pl...hs.hs.h......s...Fp.p.hhp..hhsp.@hc.h+.h.t....
Consensus_ss:                                         hhhhhhhhhhhhhhhhhhhhh    hhhhhhhhhhh    hhhhhhhhhhhhhhhhh

Conservation:                               5   5 5   9 5   9  9         5     5  5   99    5  5  5   5 9  5
306478699|RhVA    90  DQADLIFTTGIKMGVCGSLRTMEPAGFWEVIRWARSNVGRQALETGQKVKKVEDKRAGTQTPNEVALCQIFTMQQGIMAEEVKEARAST  178
308814347|BBLV    67  DILENIASAANTAGAIQHVINMTPKDLFDLSRWVKTAAGMEAISKITTQRRLLRQGGAGRSASQVAWVNLFTQQQADYAQEKKRKMTRF  155
213496075|STV     78  EELDLALGAAVAHGVLRSMRGITFKKFFDFLNWVKTKEGKDALGETMYAQKLEQKGRGDFSIAEVALLHCFETQRNDMLRDEKDVRLKA  166
166203315|VCV-M   77  RDMSALLALGGTR-FWELPIDATLENFIDFVKWIKSPEGRDAQTAAAKKRSLQKRAVGGLSTADVAIIQMGNSMIADYQKERKKARAPI  164
Consensus_aa:         c.hp.hhsht....hh....shp...hh-h.pWh+o..G.pAb......pplbpp..ts.o.spVAhhph.p.b.s.h.p-.Kc..h.h
Consensus_ss:         hhhhhhhhh  hhhhhhhhh   hhhhhhhhhhhhhh  hhhhhhhhhhhhhhhhh      hheehhhhhhhhhhhhhhhhhhhhhh

Conservation:         5     9 5 5              5  5            5    55  9          5 55 5      9   5   9
306478699|RhVA   179  QQEIDELTRLLRLKRAEQVKVLAEIKDKYFPANIWEEPAEAERNARCWEVYSAALVTAGRPAPIK-TEAAFKLAIDAYKNFVDTEFKTR  266
308814347|BBLV   156  ERKREDLKLQLAQLDTDEAASMERLAAKYPTQVALPATMETELVAACWAAYVADCDRRGITPSAK-TNASLTEAVKHYSANVRDQILTT  243
213496075|STV    167  EEEIADLQRKIVKRREKLEEDLIATKSNYEPVSRYVGLSDYELNCKCWSLYQQFNPDK-VTAGAKPTRKQVKEAFDMYAEFVAKTNRLE  254
166203315|VCV-M  165  EEQQRHLRRELRLLDDELAATELSVKERFGCIATYEGPDNHTVSREAYKLYLEYCRENRVRAIAS-HQGGFDKAVEMFGAKVRETHFRD  252
Consensus_aa:         ppp..cLp.bl.b.c.cb..sb..h..p@.s.s.h....php.s..t@.hY.....p....s.hp.hp..hpbAhch@...V.pp...p
Consensus_ss:         hhhhhhhhhhhhhhhhhhhhhhhhhhhh    hhh  hhhhhhhhhhhhhhhhhhhh         h  hhhhhhhhhhhhhhhhhhhh

Conservation:                   5      55      9        9 5  5  9                          555       5
306478699|RhVA   267  FIRSDEHQVALRKFADERIHFLDGIGEPKKAGTFRSLLAV-LG------VEPSAQTTTHTEERNDEDDSGGESD--VARGE--------  338
308814347|BBLV   244  YCEQENVQADLTRYAREKIQSFRDSGNQGGVTRVRNLYTAATG-AAIDIPPPRAQEVPHE--------TGPVRDPIAQTGEAQLQAVAH  323
213496075|STV    255  FLKHGNVKDELQAFINAKILGYEQLGDKGKSRYLRRQLAA-MG-VEMD-----HEVESEDEEEAAEEPAGGEAE--EGEGDDPL-----  329
166203315|VCV-M  253  YLLDATRAEFIRDYYNQKVLHLDRSGEKKQAGTFRNLVVAGCGEMALDAPPI----TTF---IFDGEDSCGESA--QSGSA--------  324
Consensus_aa:         @h.p.p....lp.@hp.+l..hc..Gp...s..hRpbhhh.hG..............s...........sts.......t.........
Consensus_ss:         hhhhhhhhhhhhhhhhhhhhhhhh    hhhhhhhhhhhh h

Conservation:                    9    5  5        55         9  5       5
306478699|RhVA   339  --------EPEPEAGGGTSRPGASGRG-AGRGKGKRKVVPERRSLRSSKTGRVTDVSVQG-------AQGDGGA------SRPKHRRR  404
308814347|BBLV   324  QALTGVVIEADPDRAQGEASSGDSDRASAASTESTRRLQPDRRVKKPRTTGR-----------------------------------  375
213496075|STV    330  QATV----GDNPD---GEARGEEDTAVQVAS--------PAVTRNRGRGRGRVTPLSQRK-------DRG------------------  377
166203315|VCV-M  325  --------AGEPAAKKKRGRPAGTGKA-AARDKADKR-PPKKRGDERNPAQ---PIP-QGQEDQDTAALGDGSQPNDPNVNVPV----  394
Consensus_aa:         .........spP.....ptps..ss.s.htp........P..p..c.p.......................................
Consensus_ss:         hhh          hh           h h     h     hhh
```

Figure S3. Multiple sequence alignment of amalgaviral capsid proteins. All sequences are indicated with their GenBank identifiers followed by abbreviated virus names. Positively charged amino acid residues predicted to be involved in RNA binding are shown in bold and underlined. Residues The last two lines in each block show consensus amino acid sequence (Consensus_aa) and consensus predicted secondary structures (Consensus_ss). The protein sequences are colored according to predicted secondary structures (red: alpha-helix, blue: beta-strand). Consensus predicted secondary structure symbols: alpha-helix: h; beta-strand: e. Consensus amino acid symbols are: conserved amino acids are in uppercase letters; aliphatic (I, V, L): l; aromatic (Y, H, W, F): @; hydrophobic (W, F, Y, M, L, I, V, A, C, T, H): h; alcohol (S, T): o; polar residues (D, E, H, K, N, Q, R, S, T): p; tiny (A, G, C, S): t; small (A, G, C, S, V, N, D, T, P): s; bulky residues (E, F, I, K, L, M, Q, R, W, Y): b; positively charged (K, R, H): +; negatively charged (D, E): −; charged (D, E, K, R, H): c. The alignment was constructed with PROMALS3D (http://prodata.swmed.edu/promals3d).
